# C-Terminal p53 Palindromic Tetrapeptide Restores Full Apoptotic Function to Mutant p53 Cancer Cells In Vitro and In Vivo

**DOI:** 10.3390/biomedicines11010137

**Published:** 2023-01-05

**Authors:** Robert L. Fine, Yuehua Mao, Richard Dinnen, Ramon V. Rosal, Anthony Raffo, Uri Hochfeld, Patrick Senatus, Jeffrey N. Bruce, Gwen Nichols, Hsin Wang, Yongliang Li, Paul W. Brandt-Rauf

**Affiliations:** 1 Experimental Therapeutics Program, Division of Medical Oncology, College of Physicians and Surgeons of Columbia University, New York, NY 10314, USA; 2 Department of Environmental Health Sciences, Mailman School of Public Health of Columbia University, New York, NY 10314, USA; 3 Department of Neurosurgery, Neurologic Institute of New York, Columbia University Medical Center, New York, NY 10032, USA; 4 Department of Chemistry, College of Staten Island, 2800 Victory Boulevard, New York, NY 10314, USA; 5 School of Biomedical Engineering, Science and Health Systems, Drexel University, Philadelphia, PA 19104, USA

**Keywords:** p53, p53 peptide, apoptosis, breast cancer, Fas, Bax, ROS

## Abstract

We previously demonstrated that a synthetic monomer peptide derived from the C-terminus of p53 (aa 361–382) induced preferential apoptosis in mutant p53 malignant cells, but not normal cells. The major problem with the peptide was its short half-life (half-life < 10 min.) due to a random coil topology found in 3D proton NMR spectroscopy studies. To induce secondary/tertiary structures to produce more stability, we developed a peptide modelled after the tetrameric structure of p53 essential for activation of target genes. Starting with the above monomer peptide (aa 361–382), we added the nuclear localization sequence of p53 (aa 353–360) and the end of the C-terminal sequence (aa 383–393), resulting in a monomer spanning aa 353–393. Four monomers were linked by glycine to maximize flexibility and in a palindromic order that mimics p53 tetramer formation with four orthogonal alpha helices, which is required for p53 transactivation of target genes. This is now known as the 4 repeat-palindromic-p53 peptide or (4R-Pal-p53p). We explored two methods for testing the activity of the palindromic tetrapeptide: (1) exogenous peptide with a truncated antennapedia carrier (Ant) and (2) a doxycycline (Dox) inducer for endogenous expression. The exogenous peptide, 4R-Pal-p53p-Ant, contained a His tag at the N-terminal and a truncated 17aa Ant at the C-terminal. Exposure of human breast cancer MB-468 cells and human skin squamous cell cancer cells (both with mutant p53, 273 Arg->His) with purified peptide at 7 µM and 15 µM produced 52% and 75%, cell death, respectively. Comparatively, the monomeric p53 C-terminal peptide-Ant (aa 361–382, termed p53p-Ant), at 15 µM and 30 µM induced 15% and 24% cell death, respectively. Compared to the p53p-Ant, the exogenous 4R-pal-p53p-Ant was over five-fold more potent for inducing apoptosis at an equimolar concentration (15 µM). Endogenous 4R-Pal-p53p expression (without Ant), induced by Dox, resulted in 43% cell death in an engineered MB468 breast cancer stable cell line, while endogenous p53 C-terminal monomeric peptide expression produced no cell death due to rapid peptide degradation. The mechanism of apoptosis from 4R-Pal-p53p involved the extrinsic and intrinsic pathways (FAS, caspase-8, Bax, PUMA) for apoptosis, as well as increasing reactive oxygen species (ROS). All three death pathways were induced from transcriptional/translational activation of pro-apoptotic genes. Additionally, mRNA of p53 target genes (Bax and Fas) increased 14-fold and 18-fold, respectively, implying that the 4R-Pal-p53p restored full apoptotic potential to mutant p53. Monomeric p53p only increased Fas expression without a transcriptional or translational increase in Fas, and other genes and human marrow stem cell studies revealed no toxicity to normal stem cells for granulocytes, erythrocytes, monocytes, and macrophages (CFU-GEMM). Additionally, the peptide specifically targeted pre-malignant and malignant cells with mutant p53 and was not toxic to normal cells with basal levels of WT p53.

## 1. Introduction

**Figure 1:**
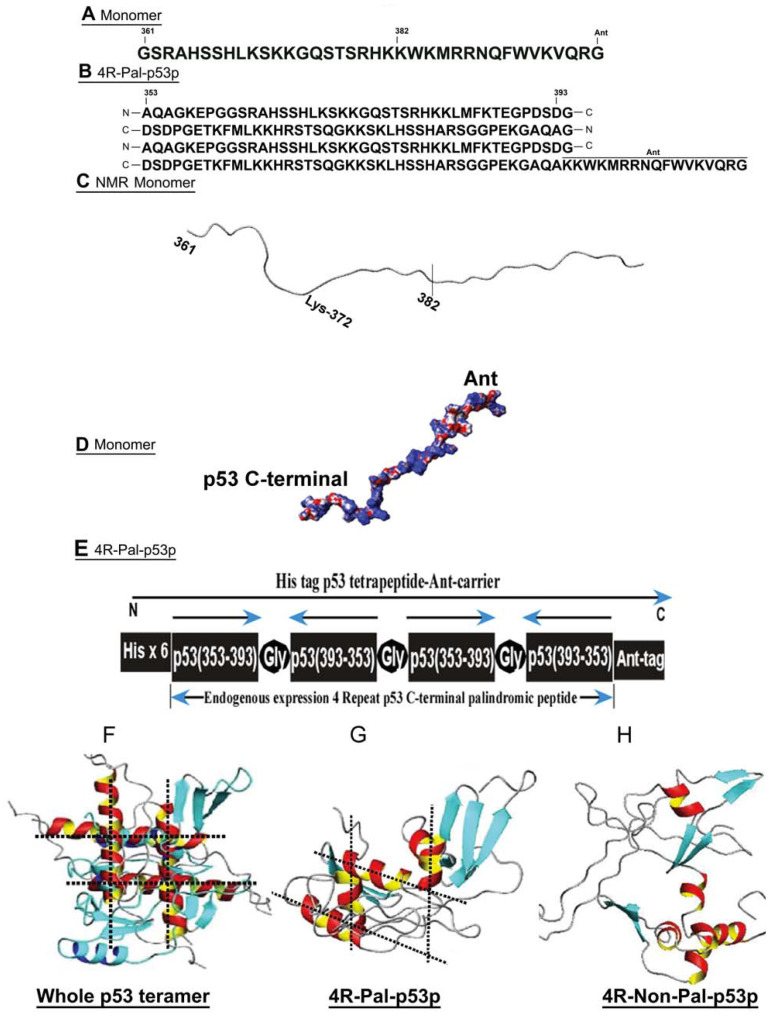
Proton-NMR derived structure of the monomeric p53p, and predicted structure of C-terminal p53 tetrapeptides. (**A**) The aa sequence structure of p53-Ant monomer. (**B**) The aa sequence structure of the 4 repeat palindromic tetrapeptide. (**C**) The ionic surface area of the p53-Ant monomer peptide. (**D**) Space filling model showing amphipathic structure formation. Blue (positive), grey (neutral), and red (negative). (**E**) The order and arrangement of the palindromic tetrapeptide. The tetrapeptide was expressed endogenously via stable transfection with plasmid or transient transfection with Ad 5 vector without 6His and Ant. The 4R-Pal-p53p with 6His and Ant was synthesized and purified for exogenous exposures. (**F**) The whole p53 tetramer structure. (**G**) The predicted structure of the 4R-Pal-p53p endogenously expressed protein without Ant. (**H**) The predicted structure of the non-palindromic 4R-NonPal-p53p peptide.

p53 plays a pivotal role in suppressing tumorigenesis by inducing cell cycle arrest, apoptosis and DNA repair. More than 50% of human cancers lack functional p53 because of mutation, deletion or inactivation. Since p53 is vital in controlling cell growth, it is an ideal target for the design of novel treatments for cancer. An emerging area for novel treatments for cancer is peptide therapeutics that target mutant p53 and through humeral and cell based immunotherapies [1] For example, we previously showed that a C-terminal p53 synthetic peptide (aa 361–382) fused at its C-terminus to the truncated 17 amino-acid intracellular transfer domain of *Drosophila* homeobox protein Antennapedia (Ant) (Figure 1A) induced p53-dependent, Fas-mediated apoptosis in breast cancer cell lines with endogenous p53 mutations or overexpressed wild-type p53 [2,3,4] The mechanism was solely through re-distribution of Fas to the extracellular space without new protein synthesis.

However, one problem of peptide-derived therapeutics is their short half-life due to rapid proteolysis in serum and the cell. Other investigators who have tested variants of the C-terminal p53 peptide as a cancer therapeutic [5,6,7,8,9] have also noted its limited potential because of its short half-life due to rapid proteolysis. A report using a synthetic D-amino acid version of our p53 peptide in an inverse configuration (aa 361–382) significantly increased the peptide’s half-life and has been shown to increase survival in a mouse malignant ascites model. However, it is costly to synthesize in large quantities, and its mechanisms of apoptosis were not investigated [7,10]. In addition, its inability to be catabolized may lead to unknown, prolonged effects in normal cells. We and other investigators have also not been able to demonstrate that p53 peptides actually transactivate p53 target genes for apoptosis in situ rather than just increasing the expression or activation of pre-existing effector molecules without increasing gene transcription–translation of apoptotic effectors. Therefore, if these levels of pre-existing effectors are insufficient or have a defective pathway for apoptosis (i.e., Fas), the cancer cell may be resistant.

These deficiencies have led others to investigate small molecules that mimic the effect of the peptide, such as PRIMA-1 [11] and a small molecule antagonist of MDM2 [12]. However, small synthetic molecules have not demonstrated an ability to transactivate multiple p53 target genes for apoptosis in situ or to transactivate in vivo target genes for p53 other than in an in vitro reporter construct for p21. Thus, these have not yet been shown to be ideal for reliable induction of p53 target genes for apoptosis because of a short half-life, chemically related toxicities, in vivo instability and the need to have endogenous WT p53 for inhibitors of MDM-2.

Our hypothesis is that if peptide-based p53 molecules had longer half-lives and could induce multiple pathways for apoptosis (e.g., through transactivation of p53 target genes for intrinsic and extrinsic pathways), they could have greater potential utility as therapeutic agents for mutant p53 cancers. The effectiveness of the p53-Ant peptide as a therapeutic agent was found by us to be limited by its rapid degradation due to its lack of structure based on computational studies. In an attempt to construct a more stable and more active peptide, we studied characteristics of the whole p53. The tetramerization formation of p53 is critical for its ability to transactivate target genes. Since we, as well as others, have shown that the p53-Ant peptide binds to p53, we hypothesized that modification of the C-terminal peptide (aa 361–382) could act as a scaffold for tetramerization that is lost in mutant p53. We designed, through computational prediction modelling, a four repeat of this monomer peptide in a palindromic order to simulate the p53 tetramer structural organization [13]. This palindromic four repeat is very stable in serum and in situ (half-life = 66 h), while the monomeric p53p had a half-life in serum of <10 min when applied exogenously. We believe that the 4R-Pal-p53p acts like a scaffold to bind four mutant p53 molecules and sterically converts them to a WT phenotype. To test the hypothesis of improved activity with this peptide, we generated stably transfected Tet-On MDA-MB468 cells (p53, R273H) which inducibly express the endogenous p53 palindromic tetrapeptide of aa 353–393, and we produced the same peptide with Ant in a bacterial expression system for exogenous exposure experiments. The exogenous peptide, 4R-Pal-p53p-Ant, contained aa 353-393-Gly 393-353-Gly 353- 393-Gly 393-353 with a N-terminal His tag for purification and a C-terminal, truncated 17aa Ant carrier. The endogenously expressed peptide, under a Tet-On inducer, was similar, but did not contain the His tag and Ant carrier moieties.

## 2. Materials and Methods

### 2.1. Plasmid Construction

**pTRE-GFP construction:** To generate the control construct with GFP under the control of the tetracycline-responsive element (Tet-On), a DNA fragment corresponding to GFP sequence of pEGFP-N2 (Clontech Laboratories, Inc., Mountain View, CA, USA) was amplified by PCR and inserted in the multiple cloning sites (MCS) of pTRE2hyg (Clontech Laboratories, Inc.), yielding pTRE-GFP.

**pTRE-p53pAnt-GFP construction (monomer):** DNA fragment corresponding to p53 aa 353 to 393 and antennapedia (Ant) was amplified by PCR with corresponding primer, which included a Kozak consensus sequence, start codon and inserted in frame to upstream GFP of pTRE-GFP, yielding pTRE-p53p-GFP, pTRE-p53-Ant-GFP.

**pTRE-4R-Pal-p53p construction:** DNA fragment corresponding to p53 aa 353 to 393 was amplified by PCR with primers as mentioned above. Then, using PCR with series reverse primers corresponding to p53 aa 393 to 353, the DNA fragment was extended from 3′ to produce a two repeat DNA fragment as p53 353-393-G-393-353. We inserted an extra glycine codon in between each fragment repeat to maximize flexibility of the peptide. Then, the two repeats were cut by restriction enzyme MspI and ligated to 4 repeats as p53 N-353-393-G-393-353-G-353-393-G-393-353-C. The 4 repeat of p53p was created with a restriction site (BamHI/ClaI) and inserted into the corresponding sites in the MCS of pTRE2hyg, yielding pTRE-4R-Pal-p53p. Based on the above constructs, we replaced the Tet-On response element with a CMV promoter and constructed monomeric pCMVp53p, pCMVp53pAnt, pCMVp53pHis, pCMVp53pGFP, pCMVp53pAntGFP, and palindromic pCMV4R-Pal-p53p plasmids with all these containing aa 353-393 of p53. Using the same techniques, we also constructed non-palindromic controls: pTRE-4R-NonPal-p53p and pCMV-4R-NonPal-p53p, which are 4-repeats of p53 aa N-353-393-G-353-393-G-353-393-G-353-393-C.

**pQE-6xHis-4R-Pal-p53p-Ant construction:** Using the same principle, we created 6xHis-4R-Pal-p53p-Ant fragments and restriction sites EcoRI/HindIII inserted into the same sites of pQE-60 (Qiagen, Valencia, CA, USA), yielding pQE-6xHis-4R-Pal-p53p-Ant for production of exogenous tetrapeptides (Figure 1B,E). All constructs above were DNA sequenced to verify correct sequence structure.

### 2.2. Dominant Negative FADD Plasmid Construction

Ad-DN-FADD was previously constructed in our lab [14] and 50 MOI was used.

### 2.3. Expression and Purification of His-4R-Pal-p53p

According to the manufacturer’s instructions (Qiagen, Valencia, CA, USA), the purified protein was then dialyzed in PBS and concentrated by Centriprep YM-10 (Millipore). The protein concentration was measured spectrophotometrically at 280 nm or by using a dye-binding assay.

### 2.4. Cell Culture and Generation of Inducible Lines

MB468 (breast, p53 R273H) and A431 (skin, p53 R273H) cells were cultured as instructed by ATCC. All 22 cell lines and their accession numbers were obtained from ATCC (Table 1). MB468 cells were transfected with pTet-On (Clontech Laboratories, Inc., Mountain View, CA, USA) using calcium phosphate precipitation and selected by 400 µg/mL G418 (Sigma, St. Louis, MO, USA). Expression of the Tet-On transactivator in G418 resistant cell lines was determined by a luciferase assay (pTRE-Luc; Clontech Laboratories, Inc., Mountain View, CA, USA, and Dual-Luciferase Reporter Assay System; Promega, Madison, WI, USA). To establish Dox-inducible lines, a single stable cell line, with the highest luciferase activity after induction (22 fold induction by Dox), was transfected with one of the following plasmid: pTRE-4R-Pal-p53p, pTRE-p53p, pTRE-p53GFP, pTRE-p53AntGFP, pTRE-AntGFP and pTRE-GFP. After selection with 400 µg/mL G418 and 200 µg/mL hygromycin, resistant clones were treated with 2 µg/mL Dox for 24 h. The cell lysates were analyzed by immunoblotting.

### 2.5. Flow Cytometric Analysis for Sub-G1 Cell Cycle and TUNEL Staining

MB468 inducible stable cells were treated with Dox for various time points, and all cells were collected and analyzed for apoptosis with PI staining, TUNEL and annexin V assay. The procedure was described in our previous paper [15].

### 2.6. Analysis of Fas and Bax Gene Expression

Total RNA was extracted from Dox inducible 4R-Pal-p53p MB468 cells stably transfected with 4R-Pal-p53p after a 24 hr incubation with Dox. The levels of Fas and Bax mRNA expression were measured using a LightCycler RNA Amplification SYBR Green I real-time reverse transcriptase–polymerase chain reaction (real-time-RT-PCR) assay on a LightCycler (Roche Diagnostics, Basel, Switzerland). The relative quantities were calculated using standard curves generated from known dilutions of cDNA from untreated 4R-Pal-p53p MB468 stable cells and normalized to endogenous *GAPDH* mRNA levels.

### 2.7. Immunocytochemistry

To study the localization of 4R-Pal-p53p, which contains the C-terminal nuclear localization sequence of p53, MDA-MB468 cells were transiently transfected with pCMV-4R-Pal-p53pGFP or pCMV-GFP as a control by FuGENE 6 (Roche) for 24 h. Cells were then fixed in 4% paraformaldehyde in PBS for 10 min. For the localization of p53, monoclonal anti-p53 (DO-1, aa 21 to 25, Santa Cruz) was used at a 5 μg/mL concentration, the DO-1 Ab will detect endogenous mutant p53, but not the p53 peptide. TR conjugated goat anti-mouse antibody (Santa Cruz Biotechnology, Dallas, TX, USA) was used as a secondary antibody. The coverslips were mounted in Vectashield mounting media with DAPI (Vector Laboratories Inc., Burlingame, CA, USA). Fluorescent images were obtained using a Zeiss epi-fluorescent microscope (Axiovert 200M).

## 3. Results

### 3.1. NMR and Predicted Structures of p53 Peptides

The results of the proton NMR studies indicated that the p53-Ant monomer is primarily a random coil structure, as previously predicted, with a minor coiled loop region around Lys 372 (Figure 1C). Figure 1D depicts the Ionic Surface Area of the p53-Ant monomer peptide: Blue (Positive), Grey (Neutral), and Red (Negative). The topology of the peptide by structural prediction and proton NMR suggested that there was no tertiary structure, only a secondary flexible string structure.

### 3.2. Effect of Exogenous 4R-Pal-p53p-Ant vs. Monomer p53p-Ant on MB468 Cells

**Figure 2:**
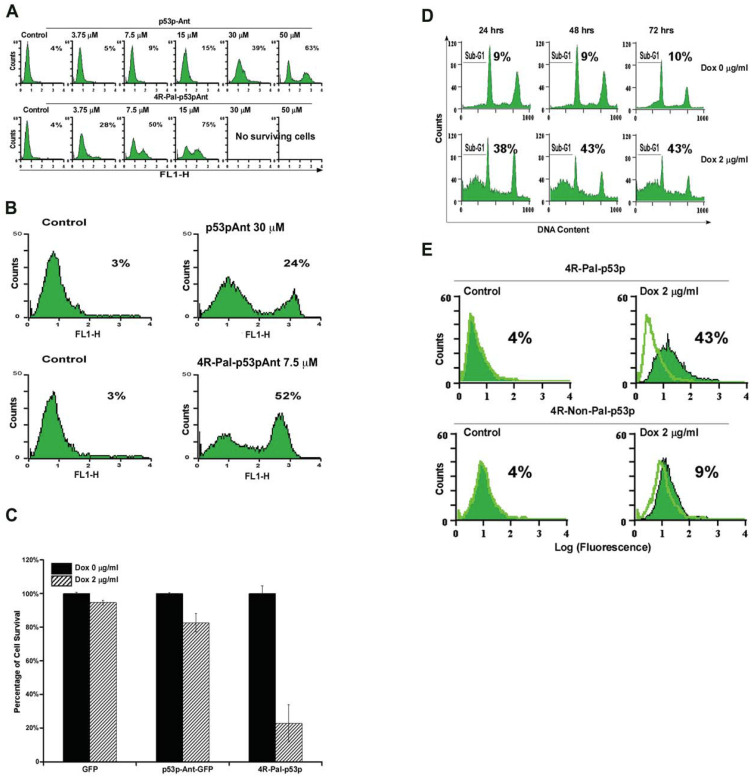
(**A**) **Effect of exogenous 4R-Pal-p53p-Ant in cancer cell lines.** MB468 human breast cancer cells (mutant p53 R273H) were exposed for 6 h to control (no peptide) or to either the monomeric p53pAnt (aa 361–382) or to the His tagged 4R-Pal-p53p-Ant. Annexin V staining was assayed by flow cytometry. The percent within each graph represents the percent of apoptotic cells. At concentrations ≥30 μM 4R-Pal-p53p-Ant produced 100% cell kill. (**B**) **A431 Human squamous carcinoma cells (mutant p53 R273H) treated with either p53pAnt or 4R-Pal-p53pAnt**. 4R-Pal-p53pAnt (7.5 μM) was 8.7 fold more potent on an equimolar basis by Annexin V assay for inducing apoptosis than p53pAnt (30 μM) when exogenously delivered. Similarly, endogenous expression of 4R-Pal-p53p produced 43% cell kill while endogenous expression by a Dox inducible promoter for monomeric p53p-Ant-GFP had no significant cell death. (**C**) **Effect of endogenous, regulated expression of the 4R-Pal-p53p on cell viability.** MB468 cells with mutant p53 (R273H), engineered to express GFP or p53p-Ant- GFP or 4R-Pal-p53p under the control of a Tet-On promoter, were cultured without or with 2 μg/mL Dox for 48 h and analyzed by trypan blue. The tetrapeptide and monomeric p53p produced 80% and 15% apoptosis, respectively. (**D**) **Effect of endogenous expression of the 4R-Pal-p53 on apoptosis**. MB468 cells with mutant p53 (R273H), were engineered to express the 4R-Pal-p53p under the control of a Tet-On promoter, were exposed to 2 μg/mL Dox for 24, 48 or 72 h. Cells were collected, fixed in ice cold 70% ethanol, and the DNA was stained with PI. Cell cycle profiles were obtained by flow cytometry, and the percentages of sub-G1 cell particles, indicative of apoptosis, were quantified. Endogenously expressed peptides did not contain the Ant sequence and Dox induced 38% sub G1 particles at 24 h and 43% sub-G1 particles at 48 and 72 h. (**E**) **Comparison of Dox induced endogenous expression of palindromic (4R-Pal-p53p) and non-palindromic (4R-NonPal-p53p) peptide in MB468 cells breast cancer cells with mutant p53 (R273H).** Non-palindromic and palindromic 4R-Pal-p53p induced 9% and 43% Annexin V positive cells, respectively.

To test the effect of exogenously purified His-4R-Pal-p53p-Ant in the culture we exposed MB468 breast cancer cells to titrations of 4R-Pal-p53p-Ant or to equimolar amounts of monomeric p53p-Ant for 6 h. Apoptotic cells were determined by annexin V assay. The results are shown in Figure 2A, 4R-Pal-p53p-Ant was 5 fold more effective for inducing apoptosis than monomeric p53p-Ant at 15 µM; 75% versus 15%, respectively. The IC50 for p53p-Ant was 5.3 fold higher compared to 4R-Pal-p53p-Ant (40 µM vs. 7.5 µM). In the A431 cell line (p53, R273H) [16], 4R-Pal-p53p-Ant induced 8.7 fold more apoptosis than the equimolar monomeric p53p-Ant in annexin V assays (Figure 2B). Addition of 30 µM 4R-Pal-p53p-Ant to non-malignant breast cell lines with WT p53 (MCF 10-2A and MCF 10F) showed no cell death above saline control (data not shown). Similarly, the 4R-NONPal-p53p-Ant was at least nine-fold less potent than the palindromic 4R-Pal-p53p-Ant in inducing apoptosis (data not shown).

### 3.3. Endogenous 4R-Pal-p53p Expression in Tet-On Inducible MDA-MB468 Cells

We induced high expression of the palindromic tetrameric peptide without Ant in Tet-On inducible MDA-MB468 cells using the highest fold-inducible clones. Additionally, we used the same inducible clones to express several control peptides, including GFP and monomers of p53pGFP. The anti-p53 antibody PAb-421, epitope aa 371–380 was utilized to measure expression of the peptides via immunoblotting after 48 h. exposure to Dox. Cell death was measured by trypan blue exclusion and PI staining in flow cytometry. By trypan blue exclusion, Dox exposure to engineered MB468 cells expressing endogenous 4R-Pal-p53p killed over 80% of the cells. Whereas the same MB468 cells expressing either GFP or p53p-Ant-GFP from Dox exposure as a control showed approximately 5% and 20% cell death, respectively (Figure 2C). However, MB468 cells expressing 4R-Pal-p53p showed 70% cytotoxicity by trypen blue (Figure 2C). By PI staining, MB468 cells expressing 4R-Pal-p53p induced 43% cell death (Figure 2D). In annexin V assays, cells expressing 4R-NonPal-p53p induced 9% apoptosis whereas the 4R-Pal-p53p induced 43% cell death (Figure 2E). Importantly, when p53p and p53pAnt were endogenously expressed, no toxicity above control was detected, implying rapid degradation of the peptide upon synthesis. Western blot of cells engineered to express p53p and p53pAnt upon Dox exposure did not show any stable or detectable p53 peptide expression (data shown upon request).

### 3.4. Mechanisms of Apoptosis by 4R-Pal-p53p Induction of p53 Target Genes for Apoptosis: Intrinsic and Extrinsic Pathways

**Figure 3:**
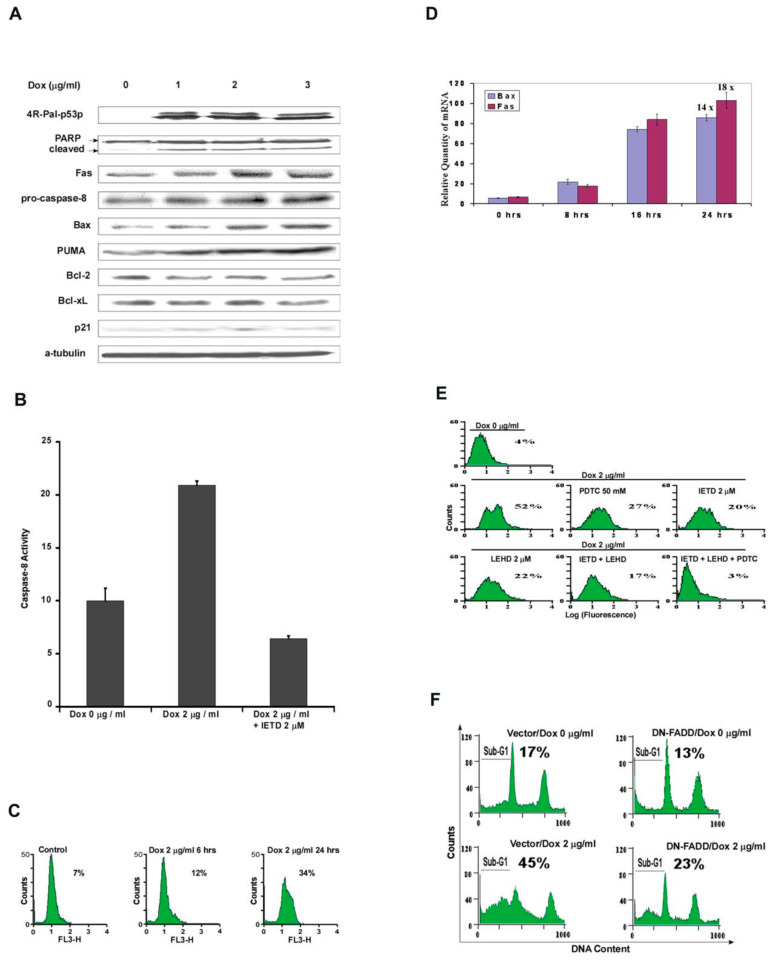
(**A**) **Changes in the protein expression levels for the pro-apoptotic p53 target genes for the intrinsic and extrinsic pathways of apoptosis**. Endogenous protein expression induced by 4R-Pal-p53p was assessed for: Fas, pro-caspase 8, PARP, Bax, PUMA, Bcl-2, Bcl-X_L_ and p21. The Western blot of engineered MB468 cells exposed to Dox shows up-regulation and translation of proteins for the extrinsic pathway (Fas, pro- caspase 8 and PARP) and intrinsic pathway of apoptosis (Bax, PUMA, Bcl-2 and Bcl- X_L_). The levels of Bcl-2 and Bcl-X_L_ decreased 50% by densitometry but there was no change in p21^WAF-1/CIP^ levels from 4R-Pal-p53p. (**B**) **Caspase 8 activity assay**. After 24 hours of exposure to Dox, caspase 8 activity doubled as compared to control. Caspase 8 inhibitor (IETD-FMK) at 2 μM decreased the basal caspase 8 activity to 55% below its control level. (**C**) **Generation of ROS by 4R-Pal-p53p**. Regulated expression of endogenous 4R-Pal-p53p by Dox in engineered MB468 breast cancer cells (mutant p53 R272H) demonstrated a 4.9 fold increase in ROS levels (mainly O_2^−^_). This was quantitated by using the probe dihydroethidium (DHE) in FACS analysis which measures ROS species, especially O_2^−^_. (**D**) **qRT-PCR for Bax and Fas**. Engineered MB468 cells were exposed to Dox (2 μg/mL) 0, 8, 16, and 24 hours. The level of Fas and Bax expression was determined by real-time qRT-PCR and normalized to GAPDH, Bax and Fas mRNA increased 14 and 18 fold, respectively. The R value curve for both Bax and Fas mRNA was equal to 1.0. (**E**) **Reversal of the apoptotic effects of 4R-Pal-p53p by inhibitors of caspases 8 and 9 and ROS**. Regulated expression of the 4R-Pal-p53p resulted in the endonuclease cleavage of chromatin DNA into oligonucleosomes (TUNEL), as seen as a shift from no Dox (control = 4%) to 24 hours after 2 μg/mL Dox (52%) (Figure 2B). ROS inhibitor (PDTC) at 50 μM, added 6 hours after Dox, decreased the TUNEL shift from 52% to 27% positivity (48% decrease). Caspase 8 inhibitor (2 μM IETD-FMK) decreased the TUNEL shift to 20% positivity (61% decrease) and caspase 9 inhibitor (2 μM LEHD- FMK) decreased the TUNEL shift to 22% positivity (58% decrease). Caspase 8 and caspase 9 inhibitors together, each at 2 μM, decreased TUNEL positivity to 17% (67% decrease). Inhibitors of Caspase 8 and caspase 9 with the ROS inhibitor PDTC together decreased TUNEL positivity to the baseline control value of 3%. This result suggested that all of the tetrapeptide effects were abrogated by inhibition of the intrinsic/extrinsic/ROS apoptotic pathways. (**F**) **Effects of dominant-negative FADD (DN-FADD) on 4R-Pal-p53p induced apoptosis**. Expression of the dominant-negative FADD (DN-FADD, aa 80-293) was tested in the engineered MB468 with mutant p53 (R273H) with a Dox inducible stable cell line for 4R-Pal-p53p. Cells were infected with 10 MOI adenovirus containing pAd/CMV/DsRed (vector) or pAd/CMV/DN-FADD (DN-FADD) for 24 h. Transfectants of the MB468 cell line were then treated with Dox 2 μg/mL for another 24 h and apoptotic cells were detected by PI staining. Representative histograms show apoptotic cell numbers relative to control. DN-FADD expression decreased the cytotoxicity of the tetrapeptide by 50%.

Changes in the protein expression of p53 responsive target genes also occurred in MB468 cells endogenously expressing 4R-Pal-p53p. This included an increase of 6-8-fold in the cleaved product of PARP, increased protein expression of Fas, pro-caspase 8, Bax, and PUMA. However, levels of Bcl-2 and Bcl-XL decreased 50%, and levels of p21WAF/CIPI did not change (data not shown). Caspase 8 activity increased over two fold and the specific caspase 8 inhibitor (FMK-IETD) completely inhibited the increased caspase 8 activity induced by 4R-Pal-p53p (Figure 3A). We assessed whether endogenous synthesis of 4R-Pal-p53p also affected the generation of reactive oxygen species (ROS). Figure 3B shows that induction of peptide by Dox increased ROS (O_2^−^_) species 4.9 fold by using the DHE fluorescence assay. Thus, the 3 major death pathways (intrinsic, extrinsic and ROS) were induced by the tetrameric palindromic peptide. In real time PCR, expression levels of mRNA for Bax and Fas increased 14 and 18 fold, respectively, after Dox exposure to engineered MB468 cells (Figure 3C). Endogenous expression of monomeric p53p-Ant did not induce mRNA or protein for Bax or Fas or caspase 8 and did not decrease Bcl-2 or Bcl-XL mRNA or protein levels (data not shown).

### 3.5. Effects of Inhibitors of the Intrinsic/Extrinsic/ROS Pathways Induced by 4R-Pal-p53p

Figure 3D shows endogenous expression of 4R-Pal-p53p produced 52% TUNEL positivity in engineered MB468 breast cancer cells. These cells showed a 4.9-fold increase in the ROS O_2_ levels (Figure 3B). The specific ROS inhibitor PDTC (pyrrolidine dithiocarbamate) at 50 µM reduced cell death to 27% (48% decrease) (Figure 3D). The specific caspase 8 inhibitor, IETD-FMK at 2 µM (IC50) reduced cell death to 20% (62% decrease) and the specific caspase 9 inhibitor LEHD-FMK at 2 µM (IC50), LEHD-FMK reduced cell death to 22% (58% decrease) (Figure 3D). The caspase 8 and caspase 9 inhibitors together reduced cell death to 17% (67% decrease). When the caspase 8 and 9 and ROS inhibitors were added together, cell death from endogenous peptide was totally reduced to control levels (3%) (Figure 3D). These three inhibitors alone or together showed no toxicity and had no significant effect upon Dox untreated cells at the same concentrations.

### 3.6. Dominant-Negative FADD (DN-FADD) and 4R-Pal-p53p Induced Cell Death

To determine whether FADD association to Fas after clustering could be pivotal for 4R-Pal-p53p induced cell death through the caspase 8 pathway segment and to corroborate the above experiments with the caspase 8 inhibitor, we modulated dominant negative FADD expression (aa 1–79 deleted) via an Ad5 adenovirus system. Engineered MB468 4R-Pal-p53p cells were transiently transfected with adenovirus containing control vector pAd/vector or pAd/DN-FADD (aa 80–293 of FADD) for 24 h with a CMV promoter. Prominent expression of DN-FADD was detected by Western blot (data not shown). Stable transfectants of MB468 cells were treated with Dox at 2 µg for 24 h and apoptotic cells were detected by PI staining. As shown in Figure 3E, DN-FADD expression decreased 4R-Pal-p53p induced apoptosis from 45% to 23% (51% decrease). This is similar to the results in the above experiments of a 62% decrease with the specific caspase 8 inhibitor IETD-FMK (Figure 3D), implying 4R-Pal-p53p induced apoptosis was partly mediated by the extrinsic Fas/FADD pathway.

### 3.7. Translocation of Mutant p53 to Nucleolus by p53 C-Terminal Peptide

**Figure 4:**
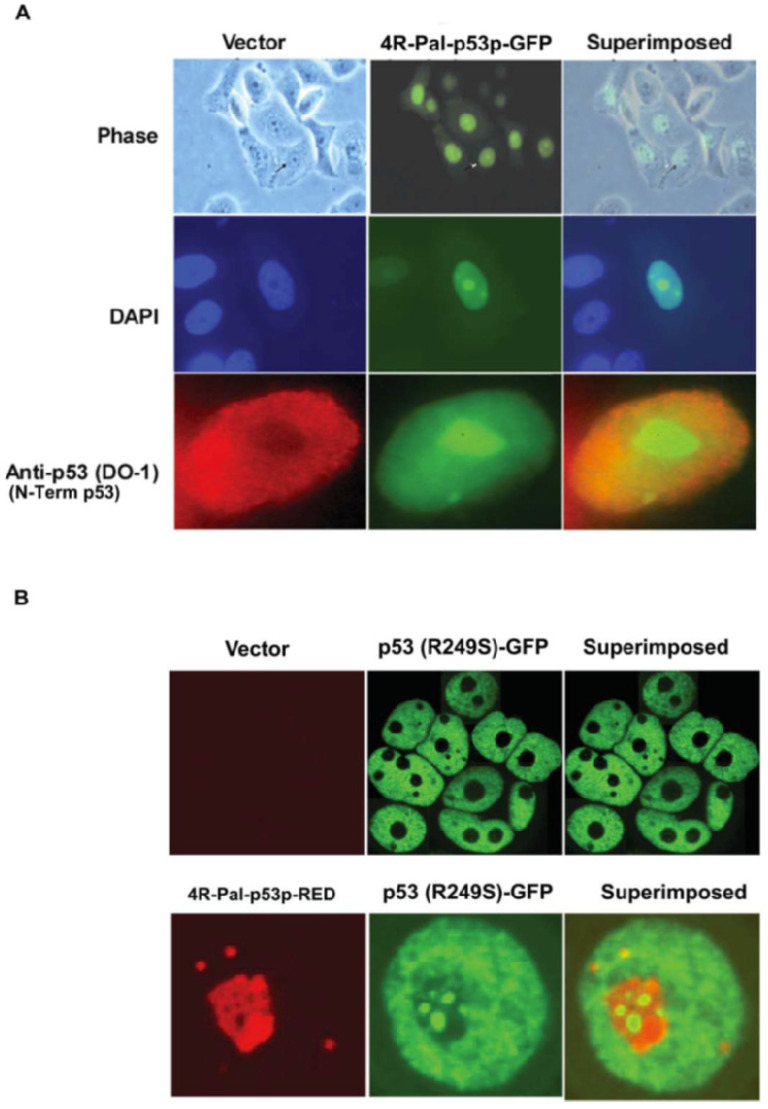
(**A**) **Subcellular localization of endogenous 4R-Pal-p53p and p53.** MDA-MB468 cells (mutant p53, R273H) were transiently transfected with Ad-4R-Pal-p53p-GFP. Twenty-four hrs after transfection, cells were fixed and labelled with a monoclonal antibody to N-terminal p53 (anti-p53 DO1 epitope aa 18–30) followed by TR labeled secondary antibody (as described in Materials and Methods). To visualize the nuclei, cells were stained with DAPI. Images were taken using an epi-fluorescent microscope. The superimposed panels were merged images of the respective images and showed that p53 localized in the nuclear and not in the nucleolus whereas 4R-Pal-p543p localize in the nuclear and nucleolus. (**B**) **4R-Pal-p53p translocates mutant p53 into the nucleolus.** H1299 (null p53) was infected with Ad-p53 (R249S)-GFP and Ad-4R-RFP (red fluorescent protein) for 48 h and followed under fluorescent confocal microscopy. This allowed us to examine whether the 4R-Pal-53p could translocate the mutant p53 (R249S) into the nucleolus. These suggested a co-localization of another type of mutant p53 with the tetrapetide into the area vital for p53 function (nucleus and nucleolus).

To characterize the 4R-Pal-p53 peptide’s localization, MDA-MB-468 cells were transiently transfected with pCMV-4R-Pal-p53pGFP and assayed by fluorescence microscopy. The endogenous basal mutant p53, stained by DO-1 (N-terminal) antibody, had the majority of its expression in the nucleus and none in the nucleolus (Figure 4A). Under phase contrast fluorescence microscope, 4R-Pal-p53p-GFP localized in the nucleus and in the nucleolus, not in the cytoplasm. By DAPI staining, most 4R-Pal- p53p-GFP was concentrated in the nucleolus. By DO-1 antibody staining, mutant p53 was found co-localized with 4R-Pal-p53p in the nucleus (Figure 4A). 4R-Pal-p53p localization in the nucleus and nucleolus suggested a possible role in RNA splicing. Additionally, Affymetrix gene array studies of Dox inducible 4R-Pal-p53p in MB468 cells with endogenous mutant p53 (R273H) demonstrated induction of multiple WT p53 target genes for RNA splicing, suggesting that the mutant p53 (R273H) ability to regulate gene splicing in the nucleolus was restored to the normal function of WT p53 by peptide (data not shown).

To further confirm whole mutant p53 co-localized with 4R-Pal-p53p, H1299 (p53 null) was co-transfected with a different form of mutant p53 (R249S)-GFP and Ad-4R-Pal-p53p-RED (red color GFP) for 24 h. Under confocal microscopy, mutant p53 alone concentrated in punctate form in nuclei and not in nucleoli, whereas in 4R-Pal-p53p treated cells, mutant p53 concentrated in the nucleus and nucleolus and co-localized with 4R-Pal-p53p (Figure 4B).

### 3.8. Interaction between 4R-Pal-p53p and p53

**Figure 5:**
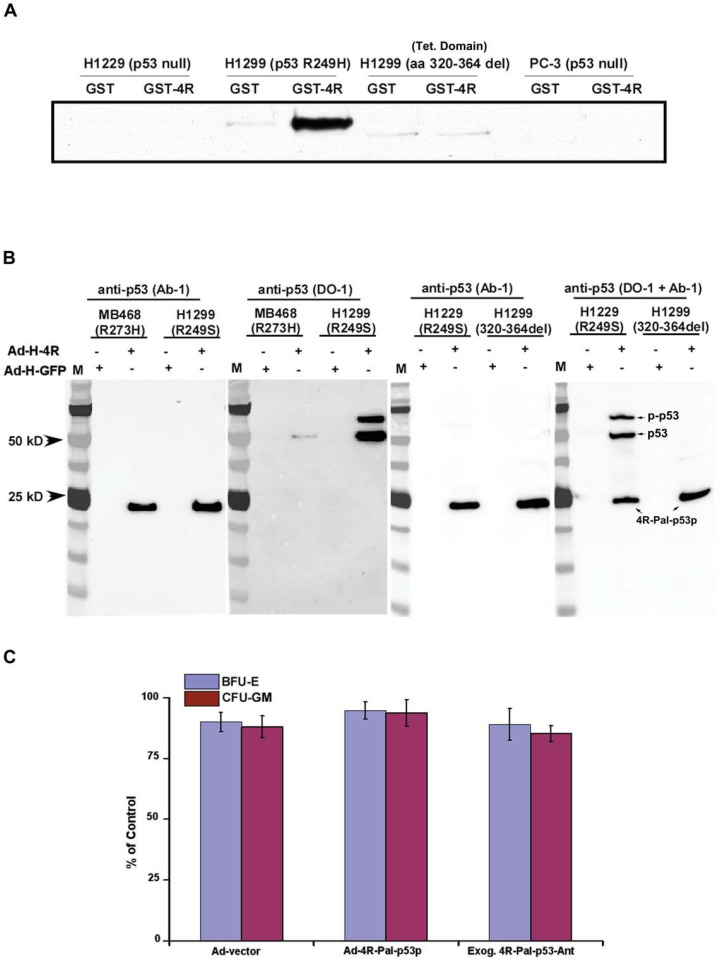
(**A**) **Co-immunoprecipitation**. This was performed to determine localization of binding to p53. It was performed with antibody to recombinant GST protein and immunoprecipitated and analyzed by Western blot with antibody to the N-terminus to p53 (DO-1 mouse IgG, epitope aa 21–25). Only GST-4R-Pal-p53p co-immunoprecipitated with mutant p53, whereas GST alone did not. This suggested that 4R-Pal-p53p binds to mutant p53. (**B**) **Determination of binding site of peptide to p53.** To determine where the 4R-Pal-p53p binds to p53, H1299 stable cell lines with p53 (R249S), and WT p53 deletion mutant (tetramerization domain deletion aa 320–364) and MB648 with mutant p53 (R273H) were infected with pAd/CMV/6xHis-GFP or pAd/CMV/6xHis-4R-Pal-p53p for 24 h. Co-precipitation was performed with nickel columns to recombinant His protein. Precipitate was analyzed by Western blot with antibody to N-terminus p53 as mentioned above and C-terminus Ab-1 mouse IgG, (epitope aa 376–378). Only His-4R-Pal-p53p co-precipitated with mutant p53 (R294S; R273H), whereas His-GFP did not. The WT p53 tetramerization domain deletion mutant showed no binding to 4R-Pal-p53p. (**C**) **CFU-GEMM assay for marrow stem cells.** The human bone marrow peripheral stem cell assay for CFU-GEMM (granulocytes, erythroid, monocyte, macrophage) tested the cytotoxicity of various adenoviral containing constructs at 50 MOI. The exposure time was for 10 days. There was no statistically significant difference between the Ad-vector, Ad-4R-Pal-p53pAnt and exogenous 4R-Pal-p53pAnt treated bone marrow stem cell toxicities for BFU-E and CFU-GM (*p* ≥ 0.8). This experiment was repeated 3 times, each in triplicate.

To determine whether 4R-Pal-p53p actually binds to mutant p53, H1299 cells (null p53) cells with stably transfected mutant p53 (R249S) or tetramerization domain deletion p53 (aa 320–364) and PC-3 cells (null p53) were infected with pAd/CMV/GST or pAd/CMV/GST-4R-Pal-p53p for 24 h (Figure 5A). Co-immunoprecipitation was performed with antibody to recombinant GST protein and immunoprecipitated and analyzed by Western blot with antibody to the N-terminus to p53 (DO-1 mouse IgG, epitope aa 21–25). Only GST-4R-Pal-p53p co-immunopreciptated with mutant p53 whereas GST alone did not. This suggested that 4R-Pal-p53p physically binds to mutant p53 (Figure 5A).

To determine where the 4R-Pal-p53p binds to p53, H1299 null p53, H1299 with mutant p53 (R249S), and H1299 p53 deletion mutant (tetramerization domain deletion aa 320–364) were infected with pAd/CMV/6xHis-GFP or pAd/CMV/6xHis-4R-Pal-p53p for 24 h. Co-precipitation was performed with nickel columns for recombinant His protein. Precipitate was analyzed by Western blot with an antibody to N-terminus p53 as mentioned above and C-terminus Ab-1 mouse IgG (epitope aa 376–378). Only His-4R-Pal-p53p co-precipitated with mutant p53 (R294S; R273H), whereas His-GFP did not. The WT p53 tetramerization domain deletion mutant showed no binding to 4R-Pal-p53p (Figure 5A,B). This indirectly confirmed that 4R-Pal-p53p directly binds to p53 at its tetramerization domain where WT p53 normally binds to form a tetramer of WT p53. Mutations in human p53 in the tetramerization domain are extremely rare which allows the majority of mutant p53 forms to bind to the tetrapeptide. Conceptually, we think that the 4R-Pal-p53p may act like a scaffold for binding to four mutant p53 monomers at their tetramerization domain and restore the apoptotic function of mutant p53 back to a WT phenotype.

### 3.9. Peptide Effects on Human Marrow Stem Cells, Normal and Premalignant Cell Lines

The human CFU-GEMM assay tests for cytotoxicity to actively growing marrow stem cells for granulocyte, erythyroid, monocyte and macrophage colonies (GEMM), which contain normal levels of WT p53. There was no additional toxicity above control when the 4R-Pal-p53p was delivered by Ad5 virus (50 MOI) or by exogenous 4R-Pal-p53p-Ant peptide at 7.5 µM (average IC50 for MDA-MB468 and A431 human mutant p53 tumor cell lines). Marrow stem cells (CD34+) obtained from normal volunteers were exposed for 24 h to virus or peptide before plating in semi-solid agar for an additional 12–14 days with either peptide or Ad5 peptide vector (Figure 5C). No toxicity was detected by endogenous or exogenous 4R-Pal-p53p to CFU-GM or to BFU-E (erythroid) colonies.

Other normal or non-malignant human cell lines with WT p53 were tested with 4R- Pal-p53p by either exogenous peptide using Ant or by Adenovirus 5 vectors carrying 4R-Pal-p53p without Ant. No additional toxicity was found after peptide exposure in non-malignant cells such as breast epithelial cells (HMEC, MCF-10-2A, MCF-10F) (Table 1).

However, the peptide was cytotoxic to pre-malignant cell lines if they had endogenous mutant p53 (pre-malignant RG/C2 colon cells (R282W) and premalignant colon BR/C1 (p53 del. 262) and breast MCF-alpha5 (R273H)). If the pre-malignant lines had wt p53 then the peptide had no toxicity to these lines (colon-AA/C1, breast MCF-10F, and MCF-10-2A) (Table 1).

### 3.10. Effect of 4R-Pal-p53p in Other Cancer Cell Lines

Importantly, the tetrapeptide induced >50% apoptosis in five of the six most common mutations in p53 that occur in human cancer: R273H, R249S, R282W, R248G and R175H (Table 1). In all null p53 cell lines, the tetrapeptide had no effect (H1299, PC-3, SKOV-3).

### 3.11. Animal Studies

**Figure 6:**
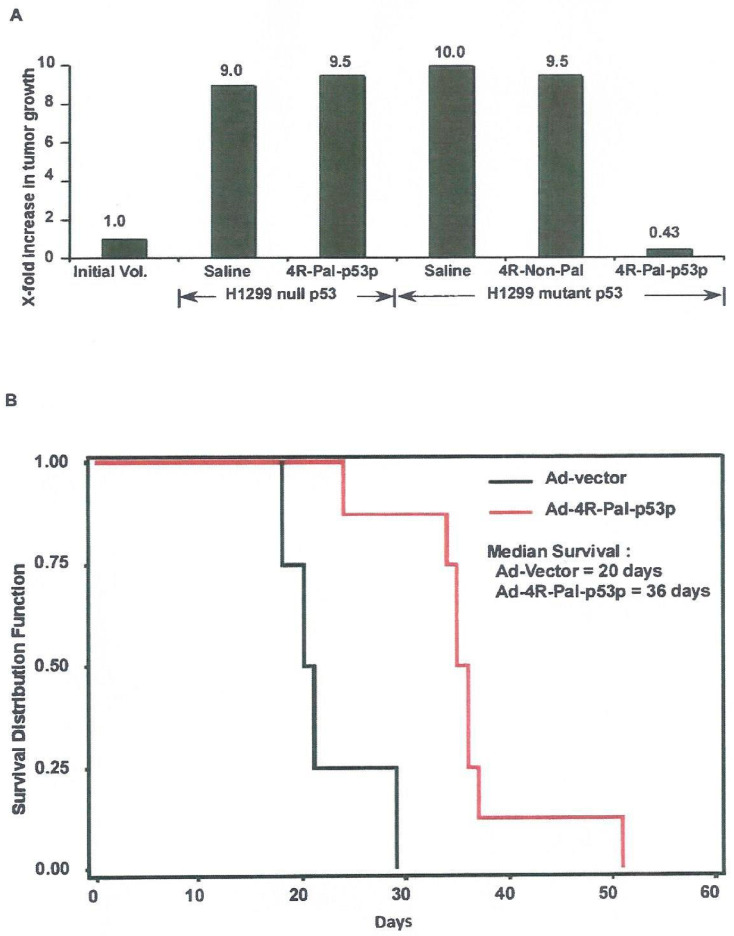
**Animal studies.** (**A**) **The effect of Ad-4R-Pal-p53p-Ant on the growth of human lung cancer xenograft tumors.** Human lung adenocarcinoma H1299 cells (null p53) and H1299 cells stably transfected with mutant p53 (R249S) were injected subcutaneously (1 × 10^6^ cells) with Matrigel into the hind flank of female athymic (nude) mice age 8–10 weeks. After approximately 10 days, when the tumors became 100 mm^3^ in size, osmotic Alzet pumps were surgically implanted juxtaposed to the tumors. The pumps delivered 100 μL over a 14 day period of an adenovirus containing the 4R-Pal-p53p plasmid. The viral titer was 1 × 10^6^ per μL, so that 1 × 10^8^ viral particles were delivered over 14 days. The volumes of the tumors were regularly monitored, and the results after 14 days of treatment are represented. Volumes were determined as the product of tumor W × L^2^ ÷ 2. Student *t*-test analysis (n = 9/group) showed a *p* < 0.001 between palindromic tetrapeptide (0.43) and non-palindromic tetrapeptide control and saline treated groups (9.0–10.0). These numbers mean that in the H1299 p53 null group the tumor grew 9.0–9.5 fold higher than at the starting point (100 mm^2^), irrespective of treatment and in the H1299 mutant p53 (R249S) group, and the saline and non-palindrome groups grew 9.5–10.0 fold larger than the starting point. However, in the tetrapeptide group, the tumors not only did not grow above starting point, but decreased 57% (end size = 0.43), implying cytotoxicity and not just cytostasis. (**B**) **Syngeneic rat glioma model.** The syngeneic, orthotopic rat glioma model 9L (mutant p53 R273H) in Fisher rats was utilized as we previously described [17,18]. The animal group treated with Ad-4R-Pal-p53p (n = 9) had longer survival times (median survival = 36 days) than the control group treated with Ad-vector (median survival = 20 days). The saline alone group (n = 9) had a median survival of 19.0 days (data not shown). Kaplan–Meier survival analysis showed a highly significant difference between the Ad-peptide and Ad control groups (Log-Rank X^2^ = 11.09, *p* = 0.0009). This translated to an 180% increase in median survival for the Ad-4R-Pal-p53p treated group in this rat model for syngeneic brain tumors.

In a subcutaneous xenograft model, lung cancer H1299 cells (null p53) and H1299 cells with stably transfected mutant p53 (R249S) were injected subcutaneously (1 × 10^6^ cells) with Matrigel into the hind flank of female athymic (nude) mice aged 8–10 weeks. After 10 days, when the tumors became visible and palpable (100 mm^3^), osmotic Alzet pumps were surgically implanted juxtaposed to the tumors. The pumps delivered 100 µL containing 1 × 10^6^ per µL Ad-4R-Pal-p53p virions over a 14 day period (total virions = 1 × 10^8^ over 14 days). The result was determined as the product of tumor W × L^2^ ÷ 2. Student T-test analysis (n = 9/group) showed statistically significant differences in tumor size (*p* < 0.001) between palindromic tetrapeptide treated-virus (0.43 mm^3^), non-palindromic tetrapeptide control (9.5 mm^3^), saline control treated groups in H1299 mutant p53 (10.0 mm^3^), H1299 null p53 group (9.0 mm^3^) and non-palindromic tetrapeptide (9.5 mm^3^) (Figure 6A). The numbers in the graph represents the x-fold increase of volume tumor growth divided by the initial volume at the start of the experiment (1.0). Thus, the 0.43 in the palindromic tetrapeptide treatment in the H1299 mutant p53 tumors implies that this group decreased its end volume of tumor from the beginning 100 mm^3^ by 57%. Conversely, the 9.5 in the non-panlindromic tetrapeptide group means the tumor volume increased 9.5 fold over the start volume of 1.0 (100 mm^3^) (Figure 6A).

In a syngeneic mutant p53 9L glioma orthotopic model in rats, transient intracerebral tumor delivery by direct intratumoral convection enhanced delivery (CED, also known as clysis) was tested. Ad-4R-Pal-p53p intratumoral delivery increased median survival by 180% over control Ad vector carrier (36 vs. 20 days). Delivery of the adenovirus into the rats was started when the tumors grew for 10 days and the tumors were at least 10 mm^3^ in volume (day 0) as shown in our previous control experiments [17,18]. The delivery of the vector was infused over 3 h/day on days 1–10 (after day 0) by the CED method. The Kaplan–Meier analysis showed statistically significant differences from treatment (n = 9) and control vector (n = 9) for survival (*p* = 0.0009) (Figure 6B).

## 4. Discussion

The monomer p53p-Ant peptide, added exogenously, induced a p53 dependent, Fas-FADD/APO-1 mediated apoptosis through interaction with the N- terminal domain of FADD [3]. This peptide is known to bind to mutant p53 and reactivate its DNA binding ability in vitro, but without transcriptional/translational syntheses for apoptotic genes [3] This suggested that this peptide may also bind to or interact with the FAS-FADD/APO-1 complex, normally inactivated when p53 is mutated, to cause an immediate type of Fas related apoptosis. The p53p-Ant monomeric peptide exhibited little structure in an aqueous extracellular-like environment by proton-NMR studies, as shown in Figure 1, and this structure changed negligibly in a membrane mimetic environment 1by ^1^H-NMR studies (Figure 1). The overall random coil structure (Figure 1) of the p53-Ant monomeric peptide may allow it to function as a molecular crutch to overcome the inactive mutant p53 structure by competitively inhibiting the inhibitory C-terminal region of p53 (360–393) and restore partial function to various forms of mutant p53. However, endogenous expression of the monomeric p53p with and without Ant, could not be detected in Western blots, implying rapid degradation. If GFP was added to its C-terminus, then expression was detectable by Western blot, implying that the large GFP molecule with tertiary structure increased its stability and half-life. However, the endogenous expression of the monomer p53p with and without Ant or GFP did not induce apoptosis in the MB468 breast or A431 squamous cancer cells. The only apoptosis induced either by p53p monomer occurred when 30 µM p53p-Ant was exogenously added to cells, which induced the extracellular membrane expression of Fas without inducing Fas or Bax transcription or translation. This induction of apoptosis was short lived due to an approximate half-life of the peptide (<10 min). No further killing of tumor cells occurred after the first 10 min of exposure, and it was tested up to 96 h after exposure.

Since the palindromic form of the 4R-Pal-p53p peptide displayed a peptide tertiary structure by predicted structural analysis, we assumed that it should be more stable with a prolonged half-life. Importantly, the alpha helical components of the predicted structure for the palindromic peptide orient orthogonally to one another (Figure 1G), similar to the overall structure of the tetramerization domain of p53 (aa 326–353) (Figure 1F), but unlike the 4R-Non-Pal-p53p (Figure 1H). Thus, it is possible that this peptide could function as a scaffold backbone capable of binding four mutant p53 molecules and promoting transcriptional activation of p53 target genes in the intrinsic and extrinsic apoptotic pathways and activation of ROS pathways for rapid p53 mediated apoptosis. These studies also suggested that there is an interaction between the C-terminus of p53 (aa 353–393) and other sections of p53 that may induce activation or suppression of p53. We have found, through deletion mutants of WT p53, that the binding of the tetrameric p53 peptide is completely abrogated when the tetramerization domain (p53 aa 326–353) is deleted in surface plasma resonance BIACORE studies (data not shown).

Both p53p monomer and the 4R-Pal-p53p did not induce p21^WAF/CIPI^ expression, which is unusual. Prior studies of small molecular modulators designed for mutant p53 have commonly shown induction of p21^WAF/CIPI^ [10,19]. p21^WAF/CIPI^ has been shown to increase drug resistance in tumors by inducing cell cycle arrest at G1/S or G2/M, which antagonizes the effects of chemotherapy [10]. Preliminary experiments have shown that p53p and 4R-Pal-p53p synergizes potently with cell cycle active chemotherapy for inducing apoptosis (data not shown). The majority of all chemotherapy agents induce apoptosis to a higher degree in actively growing cells than static, G0 cells. Thus, conceptually, to induce cell death more effectively, p21^WAF/CIPI^ levels should ideally not be increased by these peptides.

All of these findings support a restoration of apoptotic function by the palindromic tetrapeptide in these 2 mutant p53 cell lines (MB468 and A431) as well as the 5 of 6 most common mutant p53 forms in human cancer and the remaining 11 other cell lines with various forms of mutant p53. The mechanism of apoptosis was through the intrinsic and extrinsic pathways, (increased PUMA, Bax, Fas, caspase 8 and decreased Bcl-2 and Bcl-XL) and activation of the ROS O_2^−^_ pathway. These three apoptotic death pathways are the major mechanism by which WT p53 induces a fast and delayed apoptosis. In addition, the 4R-Pal-p53p induced substantial apoptosis in other mutant p53 cancer cell lines such as lung cancer cell line H1229 (p53 R249S), H889 (p53 C242S, data not shown); colon cancer cell line SW48 (p53 R248W); breast cancer cell lines MCF-7 (overexpressed wt p53), Hs578T (V157F); 2 neuroblastoma cell lines SK-N-H and SK-N-AS with overexpressed wt p53, but not in lines with normal levels of WT p53 or in null p53 cell lines. In contrast, the monomeric p53p-Ant only induced the extrinsic pathway via redistribution of Fas without transcriptional/translational transactivation of any intrinsic/extrinsic genes for apoptotic or any p53 target genes [3]. Additionally, Western blots did not show any change in the levels of Fas, Bax, Bcl-2, Bcl-XL, MCL-1 after exposure to the monomeric p53p-Ant (aa 361–382) [3].

The above experiments demonstrated that the 4R-Pal-p53p mediated the majority of its apoptotic effect through the intrinsic (increasing Bax and PUMA and decreasing Bcl-2 and Bcl-XL) and extrinsic (increasing Fas/pro-caspase 8) pathways. Specific inhibitors for each pathway (each at their IC50 of 2 µM) together blocked 67% of the apoptotic effect induced by the tetrapeptide. The remaining 33% was unaccounted for by blocking the intrinsic and extrinsic pathways, thus other mechanisms, such as the ROS pathway, were investigated. Experiments showed nearly a five-fold increase in ROS species (O_2^−^_) from exposure to 4R-Pal-p53p-Ant.

The specific ROS inhibitor PDTC with specific caspase 8 and caspase 9 inhibitors together totally blocked the cell death induced by 4R-Pal-p53p-Ant. WT p53, but not mutant p53, has been shown to increase ROS with or without transcriptional/translational activation, leading to induction of apoptosis through the rapid mitochondrial death pathway. If the 4R-Pal-p53p restored functional status to mutant p53, then it could possibly restore its transcriptional/translational ability to generate ROS, which could account for the remaining unexplained effect of the peptide for inducing apoptosis.

Experiments with human peripheral blood stem cells for CFU-GEMM (CD34+) showed no additional cytotoxicity above control from adenovirus delivered 4R-Pal-p53p or exogenously added 4R-Pal-p53p-Ant peptide. This is probably due to the normal basal, low levels of WT p53, which do not provide ample target levels for 4R-Pal-p53p. Our studies in surface plasmon resonance (Biacore) assays revealed the Kd for purified and partially purified nuclear extracts from mutant forms of p53 (R273H, and R249S) had over three-fold tighter binding than for WT p53 (data not shown). This difference in dissociation constants helps to explain why the peptide has preferential effects for mutant p53 forms and less toxicity to cancer cells with low levels of WT p53 or no toxicity to normal cells that have low basal levels of WT p53. Thus, the longer half-life of mutant p53, from lack or decreased Hdm-2 ubiquination and proteosomic degradation, leads to higher levels of mutant p53, which provide more targets for the peptide. This, along with the tighter binding constants, may explain why 4R-Pal-p53p has specificity for multiple types of mutant p53. In addition, the binding site for the peptide, the tetramerization domain of p53 (aa 320–353), is rarely mutated in human cancer with mutant p53, thus allowing the peptide the ability to restore a WT p53 phenotype in a large number of p53 mutant cell lines. However, the peptide can still kill cancer cells with elevated WT p53 (i.e., some breast cancer lines, see Table 1), levels similar to mutant p53 tumor cells with about 50% less efficacy than the same cell with equal amount of mutant p53 such as in PC-3 and H1299 null p53 cell lines with a stably transfected temperature sensitive mutant p53 (V143A) (data not shown).

In addition to its targeted specificity for mutant p53 tumor cells, we have shown that the peptide also induced apoptosis in immortalized, human pre-malignant breast and colon cells with mutant p53 [14]. A large number of human adenocarcinomas arise in the ductal epithelial lining of organs, which are amenable to delivery of the p53 tetrapeptide. Many of these pre-malignant lesions undergo a defined ontogeny of genetic mutations, which include mutation of p53 necessary for malignant transformation before an invasive malignancy develops. These mutant p53 pre-malignant and nascent malignant cells could be targets for 4R-Pal-p53p before malignant tumors develop, while still non-toxic to the normal surrounding cells. Examples of such treatable pre-malignant cells with a high incidence of mutant p53 (≥50%) include: (1) mammary ductal epithelia with high grade DCIS via intramammary ductal lavage; (2) pre-malignant pancreatic lesions with high grade PanIn 2/3 or main duct IPMN by ERCP; (3) pre-malignant skin lesions such as early basal cell carcinoma, leukoplakia, erythroplakia or solar keratosis via topical application; (4) Barrett’s esophagus via endoscopy; (5) intra-bronchial high grade dysplasia via bronchoscopy or inhalation; (6) high grade dysplasia or carcinoma in situ of the bladder via cystoscopy; and (7) high grade adenomatous colonic sessile polyps.

## 5. Conclusions

In studies of the types of mutant p53 sensitive to 4R-Pal-p53p, we have found, preliminarily, that the class of mutants p53 that can be restored to a WT p53 phenotype and inducing the 3 pathways of apoptosis are: (1) six of six DNA contact mutants (class I); (2) eight of eight common class II mutants that cause localized structural changes; and (3) half of eight common class III mutants (global structural changes) including the Zn^++^ binding site. Thus, the application and clinical efficacy of the peptide could be significant and its major limiting factor will be of delivery. We are in the process of elucidating the 3-D NMR and crystallographic structure of 4R-Pal-p53p in hopes of developing a synthetic mimetic that is cell permeable.

Thus, the 4R- Pal-p53p has potential as a therapy for: (1) mutant p53 cancers, (2) over-expressed WT p53 cancers and (3) treatment for cells with premalignant, mutant p53 status or carcinoma in situ. Importantly, the tetrapeptide forms the foundation for p53 peptide therapeutics and synthetic mimetics that are cell permeable and restore the three pathways for apoptosis with specificity to cells with mutant p53 or over-expressed WT p53.

## Figures and Tables

**Table 1:** Effect of Peptide on Cell Lines.

Cell Line	Accession No.	Human Origin	p53 Status	Apoptosis *
U87	HTB-14	Human glioma (GBM)	WT p53	-
U138	HTB-16	Human glioma (GBM)	R273H	+++
CCD-33Co	CRL-1539	Normal human colon cell	WT p53	-
AA/C1	CRL-1459	Pre-malignant colon	WT p53	-
RG/C2	CRL-1541	Pre-malignant colon	R282W	++
BR/C1	CRL-1790	Pre-malignant colon	p53 del 262	+
HCT116	CRL-2081	Human colon cancer	WT p53	++
LS123	CCL-255	Human colon cancer	R175H	++
SW48	CCL-231	Human colon cancer	R248W	++
MCF10-2A	CRL-10781	Normal human breast	WT p53	-
MCF10F	CRL-10718	Non-malignant breast	WT p53	-
MCF alpha 5	CRL-2834	Pre-malignant breast	R273H	++
MCF-7	HTB-22	Human breast cancer	WT p53	++
MDA-MB-468	HTB-132	Human breast cancer	R273H	+++
MDA-MB-231	HTB-26	Human breast cancer	R280K	+++
MDA-MB-157	HTB-24	Human breast cancer	G262del	+
Hs 578T	HTB-126	Human breast cancer	V157F	+
HCC1419	CRL-2326	Human breast cancer	Y220C	+
SKOV-3	HTB-77	Ovarian cancer	NULL	-
OVCAR-3	HTB-161	Ovarian cancer	R248G	++
PC-3	CRL-1435	Prostate cancer	NULL	-
H1299	CRL-5803	Lung cancer	NULL	-
SK-N-H	HTB-11	Neuroblastoma	Overexpressed WT p53	++
SK-N-AS	CRL-2137	Neuroblastoma	Overexpressed WT p53	++

* Apoptosis: + = >30%, ≤50%; ++ = >50%, <100%; +++ = 100%.

## Data Availability

Available upon request by primary corresponding author.

## References

[1] Chasov V., Zaripov M., Mirgayazova R., Khadiullina R., Zmievskaya E., Valliullina A., Rizvanov A., Bulatov E. (2021). Promising New Tools for Targeting p53 Mutant Cancers: Humoral and Cell Based Immunotherapies. Front. Immunol..

[2] Abarzua P., LoSardo J.E., Gubler M.L., Spathis R., Lu Y.A., Felix A., Neri A. (1996). Restoration of the transcription activation function to mutant p53 in human cancer cells. Oncogene.

[3] Kim A.L., Raffo A.J., Brandt-Rauf P.W., Pincus M.R., Monaco R., Abarzua P., Fine R.L. (1999). Conformational and molecular basis for induction of apoptosis by a p53 C-terminal peptide in human cancer cells. J. Biol. Chem..

[4] Senatus P.B., Li Y., Mandigo C., Nichols G., Moise G., Mao Y., Brown M.D., Anderson R.C., Parsa A.T., Brandt-Rauf P.W. (2006). Restoration of p53 function for selective Fas-mediated apoptosis in human and rat glioma cells in vitro and in vivo by a p53 COOH-terminal peptide. Mol. Cancer Ther..

[5] Almog N., Goldfinger N., Rotter V. (2000). p53-dependent apoptosis is regulated by a C-terminally alternatively spliced form of murine p53. Oncogene.

[6] Selivanova G., Iotsova V., Okan I., Fritsche M., Strom M., Groner B., Grafstrom R.C., Wiman K.G. (1997). Restoration of the growth suppression function of mutant p53 by a synthetic peptide derived from the p53 C-terminal domain. Nat. Med..

[7] Snyder E.L., Meade B.R., Saenz C.C., Dowdy S.F. (2004). Treatment of terminal peritoneal carcinomatosis by a transducible p53-activating peptide. PLoS Biol..

[8] Wang H., Li J.Z., Lai B.T., Yang X.H., Zhang C.Y., Yue W.T., Zhan X.P. (2003). Inhibitory effect of p53 with deletion of C-terminal 356–393 amino acids on malignant phenotype of human lung cancer cell line. Zhonghua Zhong Liu Za Zhi.

[9] Weisbart R.H., Miller C.W., Chan G., Wakelin R., Ferreri K., Koeffler H.P. (2003). Nuclear delivery of p53 C-terminal peptides into cancer cells using scFv fragments of a monoclonal antibody that penetrates living cells. Cancer Lett..

[10] Rokaeus N., Klein G., Wiman K.G., Szekely L., Mattsson K. (2007). PRIMA-1(MET) induces nucleolar accumulation of mutant p53 and PML nuclear body-associated proteins. Oncogene.

[11] Bykov V.J., Issaeva N., Shilov A., Hultcrantz M., Pugacheva E., Chumakov P., Bergman J., Wiman K.G., Selivanova G. (2002). Restoration of the tumor suppressor function to mutant p53 by a low-molecular-weight compound. Nat. Med..

[12] Vassilev L.T., Vu B.T., Graves B., Carvajal D., Podlaski F., Filipovic Z., Kong N., Kammlott U., Lukacs C., Klein C. (2004). In vivo activation of the p53 pathway by small-molecule antagonists of MDM2. Science.

[13] Clore G.M., Ernst J., Clubb R., Omichinski J.G., Kennedy W.M., Sakaguchi K., Appella E., Gronenborn A.M. (1995). Refined solution structure of the oligomerization domain of the tumour suppressor p53. Nat. Struct. Biol..

[14] Li Y., Mao Y., Rosal R.V., Dinnen R.D., Williams A.C., Brandt-Rauf P.W., Fine R.L. (2005). Selective induction of apoptosis through the FADD/caspase-8 pathway by a p53 c-terminal peptide in human pre-malignant and malignant cells. Int. J. Cancer.

[15] Li Y., Mao Y., Brandt-Rauf P.W., Williams A.C., Fine R.L. (2005). Selective induction of apoptosis in mutant p53 premalignant and malignant cancer cells by PRIMA-1 through the c-Jun-NH2-kinase pathway. Mol. Cancer Ther..

[16] Bureik M., Rief N., Drescher R., Jungbluth A., Montenarh M., Wagner P. (2000). An additional transcript of the cdc25C gene from A431 cells encodes a functional protein. Int. J. Oncol..

[17] Bruce J.N., Falavigna A., Johnson J.P., Hall J.S., Birch B.D., Yoon J.T., Wu E.X., Fine R.L., Parsa A.T. (2000). Intracerebral clysis in a rat glioma model. Neurosurgery.

[18] Kaiser M.G., Parsa A.T., Fine R.L., Hall J.S., Chakrabarti I., Bruce J.N. (2000). Tissue distribution and antitumor activity of topotecan delivered by intracerebral clysis in a rat glioma model. Neurosurgery.

[19] Wang W., Kim S.H., El-Deiry W.S. (2006). Small-molecule modulators of p53 family signaling and antitumor effects in p53-deficient human colon tumor xenografts. Proc. Natl. Acad. Sci. USA.

